# The impact of extreme weather on student online learning participation

**DOI:** 10.1186/s41039-022-00201-2

**Published:** 2022-07-14

**Authors:** Ezekiel Adriel D. Lagmay, Maria Mercedes T. Rodrigo

**Affiliations:** Ateneo de Manila University, Quezon City, Metro Manila Philippines

**Keywords:** CausalImpact, Learning Management System, Typhoon, Philippines

## Abstract

In March 2020, the COVID-19 pandemic forced over 1 billion learners to shift from face-to-face instruction to online learning. Seven months after it began, this transition became even more challenging for Filipino online learners. Eight typhoons struck the Philippines from October to November 2020. Two of these typhoons caused widespread flooding, utilities interruptions, property destruction, and loss of life. We examine how these severe weather conditions affected online learning participation of Filipino students pursuing their undergraduate and graduate studies. We used CausalImpact analysis to explore September 2020 to January 2021 data collected from the Moodle Learning Management System data of one university in the Philippines. We found that overall student online participation was significantly negatively affected by typhoons. However, the effect on participation in Assignments and Quizzes was not significant. These findings suggested that students continued to participate in activities that have a direct bearing on their final grades, rather than activities that had no impact on their course outcomes.

## Context of the study

The shift to online learning because of COVID-19 offered us a unique opportunity to quantify the impact of extreme weather on the online learning participation of Filipino students. In prior years, the majority of education in the Philippines, as in most countries, took place in person. While some institutions made use of Learning Management Systems (LMSs), most instruction was face to face. LMSs were repositories for materials, submission sites, or test platforms, but were typically not used to replace class time. The onset of the pandemic forced 1 billion students (UNESCO, 2021), including Filipinos, to shift to an online mode. The struggle to teach and learn online worsened when eight typhoons entered the Philippine Area of Responsibility (PAR) from October 11 to November 12, 2020 (Lalu, 2020). Two of them, Typhoons Goni and Vamco, were particularly destructive, causing widespread destruction, utilities disruptions, and loss of life. The migration of all instruction to digital platforms thus enabled us to capture a greater variety of instructional activities, data that were previously unavailable, and to use this data to study the effects of these typhoons on student learning behaviors.

## Effects of extreme weather on academic achievement

The immediate effects of extreme weather events such as severe typhoons and heat waves include property destruction, crop failure, and human casualties. On November 8, 2013, for example, Typhoon Haiyan made landfall in the Philippines. A Category 5 storm, it was one of the most powerful typhoons of all time. It displaced 4.1 million people, killed 6,000, damaged 1.1 million homes, and destroyed 33 million coconut trees, a major cash crop (World Vision, 2021). In total, Typhoon Haiyan caused damages estimated at US$5.8 billion. Typhoon Goni made landfall in the Philippines on October 27, 2020, seven months into the COVID-19 pandemic. Like Haiyan, Goni was a Category 5 storm, the strongest of 2020, with maximum sustained winds of 255 km per hour. It left 25 dead and damaged over 280,000 houses. Damage to crops, livestock, fisheries, and agriculture was estimated at P5 billion, while damage to infrastructure such as roads and bridges was estimated at P12.8 billion (International Federation of Red Cross & Red Crescent Societies, 2020a). Typhoon Vamco made landfall in the Philippines on November 11, 2020. Vamco was weaker than Goni, with maximum sustained winds at 155 km per hour (International Federation of Red Cross & Red Crescent Societies, 2020b). However, Vamco brought historically high levels of flooding in parts of the country—the worst in 45 years. The storm killed 101 people and left over P20 billion in damages to livelihoods and infrastructure.

The longer-term consequences of these events are far-reaching and complex. In the developing world specifically, limited savings among less wealthy households and the lack of social supports such as access to credit and insurance make it difficult for poorer families to recover from shocks caused by extreme weather (Groppo & Kraehnert, 2017; Marchetta et al., 2018). Parents are forced to shift their investments from their children’s schooling, e.g., uniforms, books, transportation, tuition (Joshi, 2019), instead directing their resources to recovery from the economic consequences of the typhoon’s damage (Deuchert & Felfe, 2015). Post-typhoon enrollment decreases. Parents spend less time on their children’s learning and care (Joshi, 2019). Children spend less time in school and more time helping at home. Teens and young adults who are transitioning from school to work are particularly vulnerable to these shocks. They are likely to drop out of school and join the workforce in order to mitigate the impact of extreme weather. Poor young women in particular are susceptible to being pushed into the labor market (Marchetta et al., 2018).

These necessary choices cause an immediate gap in learning that grows over time. When Typhoon Mike hit Cebu in 1990, the children whose houses suffered typhoon damage lagged 0.13 years behind in school. The lag grew to 0.27 years in 1998, 0.52 years in 2002, and 0.67 years in 2005. By the time children are 22 years old, the gap in educational attainment is approximated at one year (Deuchert & Felfe, 2015).

The work of Bernabe et al. (2021) agrees. They found that storms have a disruptive impact on education. In areas severely affected by winds, children are 9% more likely to accumulate an educational delay and 6.5% less likely to complete secondary education. Individuals severely affected by storms between the ages of 23 and 33 are less likely to complete higher education, reducing their ability to obtain regular salaried jobs.

One might ask: Is it not possible for these children and young adults to return to school to make up for these gaps? Cunha and Heckman (2007) argue that different stages of childhood are more receptive to certain types of inputs than others. Secondary language learning, for example, is best before 12. They also find that public training programs for adults that try to bridge learning gaps from childhood do not produce substantial gains for most of their participants and tend to be more costly than remediation provided at earlier ages.

In summary, the physical and economic damage wrought by extreme weather events has an adverse impact on educational achievement. The education of young children who come from economically disadvantaged homes receives less financial support and parental attention, resulting in an achievement gap that increases with time. Adolescents and young adults, on the other hand, are sometimes forced to discontinue their studies and to enter the workforce to help mitigate the effects of the event. Resuming studies after an interruption is challenging because oftentimes an optimal window for learning has passed and attempts at remediation are costly and generally produce fewer gains.

## Research questions

For this study, we ask two main research questions:

- RQ1: To what extent was student participation affected by Typhoons Goni and Vamco?
- RQ2: Was student participation able to return to pre-typhoon levels, or did the typhoons dampen participation for the rest of the post-typhoon period? If participation did return to pre-typhoon levels, how long did it take for participation to recover?

## Time series analysis in education

We use CausalImpact analysis (Brodersen et al., 2015) to analyze the ways in which student participation in an online learning environment was affected by Typhoons Goni and Vamco. CausalImpact is a type of causal inference analysis method for time series data.

### Time series analysis methods

Causal inference refers to a family of analysis methods that enable researchers to draw conclusions about the effect of a causal variable or treatment on some outcome or phenomenon of interest (Hill & Stuart, 2015). These methods have the same general approach: They take time series data prior to an interruption or intervention, create a model from this data, use the model to predict counterfactual post-intervention trends, and then compare the counterfactual against the actual data to check for differences. They differ in terms of their underlying modeling approach. Examples of these methods are as follows (Kuromiya et al., 2020; Moraffah et al., 2021):

- CausalImpact—It is developed to evaluate the impact of a market intervention using difference-in-difference to infer the causality from observational data. Under the hood, “…it builds a Bayesian structural time series model based on multiple comparable control groups (or markets) and uses the model to project (or forecast) a series of baseline values for the time period after the event.” (Brodersen et al., 2015; Nishida, 2017)
- Interrupted Time Series (ITS) Model—Uses segmented regression model with dummy variables representing the period of the intervention for evaluating the effectiveness of population-level interventions. It is simple in terms of interpreting the results. (Bernal et al., 2017)
- Prophet—A type of generalized additive model consisting of trend, seasonality, and holidays. There is no need to interpolate missing values since the model handles time series analysis as a curve fitting problem and can predict future values at a very high accuracy. (Taylor & Letham, 2018)
- CausalTransfer—An improvement to CausalImpact which estimates treatment effects from experiments spanning multiple time points by using a state-space model. The main issue with CausalImpact is that it “treats every time point as a separate experiment and does not pool information over time”; hence, one is “only able to observe the outcomes under the treatment for one time series and under the control for the treatment for another one, but not the potential outcome under control for the former and under treatment for the latter.” CausalTransfer “combines regression to adjust for confounding with time series modelling to learn the effect of the treatment and how it evolves over time” and does not assume that data is stationary. (Li & Bühlmann, 2020)

Several methods based on neural networks and deep learning have been introduced in recent years (Moraffah et al., 2021):

- Recurrent Marginal Structural Network (R-MSN)—A sequence-to-sequence recurrent neural network (RNN)-based architecture for forecasting responses to a series of planned treatments. In contrast to other marginal structural models (MSMs) which model “the potential outcomes associated with each possible treatment trajectory with the Inverse Probability of Treatment Weighted (IPTW),” which in turn is “dependent on a correct specification of the conditional probability of treatment assignment,” R-MSN directly learns “time-dependent treatment responses from observational data, based on the marginal structural modeling framework.” (Lim et al., 2018)
- Time Series Deconfounder—This method “uses a novel recurrent neural network architecture with multitask output to build a factor model over time and infer latent variables that render the assigned treatments conditionally independent” prior to performing causal inference with the aforementioned latent variables being used in place of the multi-cause unobserved confounders. To further ensure that the factor model is able to estimate the distribution of the assigned causes, “a validation set of subjects were considered in order to compare the similarity of the two test statistics.” This overcomes the problem of having to ensure that all the confounders are observed, which may lead to biased results otherwise. (Bica et al., 2020)
- Deep Sequential Weighting—It is used for estimating individual treatment effects with time-varying confounders by using a deep recurrent weighting neural network for inferring the hidden confounders using a combination of the current treatment assignments and historical information. The learned representations of hidden confounders combined with current observed data are then utilized for obtaining potential outcome and treatment predictions. For re-weighting the population, the time-varying inverse probabilities of treatment are computed. (Liu et al., 2020)

For their own study, Kuromiya et al. (2020) first considered ITS and Prophet as possible approaches. They found that ITS had weak predictive power and limited flexibility. Prophet was better than ITS at predicting future values. In determining the impact of an event, though, Prophet was more difficult to interpret. They therefore decided to use a method called CausalImpact instead. As this was the study that we emulated, we used CausalImpact as well. We were not able to consider using CausalTransfer nor any of the neural network/deep learning methods.

### Prior Studies using CausalImpact analysis

CausalImpact is a specific type of causal inference that enables researchers to estimate the impact of an intervention such as an ad campaign on an outcome variable such as additional clicks (Brodersen, 2014; Brodersen, et al., 2015). Given time series data, we first identify predictor variables, the outcome variable, and the pre- and post-intervention time segments. CausalImpact uses the pre-intervention data to model the relationship between the predictor variables and the outcome variable. It then uses the model to estimate the post-intervention counterfactual. The impact of the intervention is the difference between the counterfactual and the observed post-intervention data. While many algorithms may be used to model the counterfactual, CausalImpact made use of Bayesian structural time series models, explained in detail in (Brodersen, et al., 2015). The CausalImpact R package (Brodersen, 2014; Brodersen, et al., 2015) is publicly available at http://google.github.io/CausalImpact/CausalImpact.html.

CausalImpact was created within a commercial context and was intended for use on marketing data and clickstream traffic (Brodersen, 2014). Since its release in 2014, the method has also been used to model the effects of product modularity on bus manufacturing (Piran et al., 2017), US cyber policies on cyberattacks (Kumar et al., 2016), Arab uprisings and tourism (Perles-Ribes et al., 2018), and the performance of app store releases (Martin, 2016).

In 2020, Kuromiya and colleagues applied CausalImpact to estimate the effects of school closures on student use of the LMS Moodle and the electronic book reader BookRoll (Kuromiya et al., 2020). They performed this analysis for all courses in aggregate and for one specific English course. In their analysis, they found that student traffic in Moodle and BookRoll increased significantly during the COVID-19 pandemic. For all courses in aggregate, Moodle traffic increased by 163%, while BookRoll traffic increased by 77%. With the English course, Moodle traffic increased by 2227%, while BookRoll traffic increased by 875%. Note that Kuromiya and colleagues use the term “intervention” to refer to school closures rather than a new teaching strategy. They therefore expanded the definition of “intervention” to include external events that may affect a system, rather than deliberate actions from researchers, educators, or other persons that are intended to influence how the system behaves. In this study, we use this expanded definition of “intervention” to refer to the typhoons that affected online learning.

In 2021, Lagmay and Rodrigo began the analysis of Typhoons Goni and Vamco’s effects on student participation in online classes and published initial results at an international conference (Lagmay & Rodrigo, 2021). While this paper drew inspiration from Kuromiya et al. (2020), it differed in its choice of predictor variables. Lagmay and Rodrigo (2021) made use of teacher and non-editing teacher activity to predict student activity. In contrast, Kuromiya et al. (2020; personal communications, 26 January 2021) used number of logs per day as both the input variable and the outcome variable.

Lagmay and Rodrigo (2021) analyzed Moodle activity from September 9, 2020, to January 9, 2021. The pre-intervention period was defined as the pre-typhoon period from September 9, 2020, to October 28, 2020. The intervention period were the days disrupted by the typhoon, October 29 to November 13. Finally, the post-intervention period was November 14 to December 23, the period after the typhoon to just before the Christmas break. The paper found a statistically significant decrease in all LMS activity but a non-statistically significant difference in activities related to assessment. The paper we present here expands the Lagmay and Rodrigo (2021) paper by experimenting with the time periods.

While much educational research makes use of causal inference in general, as of the time of this writing, the works of Kuromiya et al. (2020) and Lagmay and Rodrigo (2021) were the only applications of CausalImpact on educational data that our survey of the literature could find.

## Dataset

The dataset was composed of a time series of log data from the Moodle of a privately owned university in Metro Manila, Philippines. Prior to the study, the researchers conferred with the University Data Protection Office and the University Counsel to determine whether we needed to seek informed consent from faculty and students to access their Moodle data. Since the data that we received were anonymized and because we did not have the ability to re-identify the same, there was no need to seek informed consent from the Moodle users (J. Jacob, personal communication, 25 September 2020; P. Sison-Arroyo, personal communication, 25 September 2020). Furthermore, the University Research Ethics Office determined that our research protocol was considered exempt from institutional ethics review because it was research conducted in educational settings involving normal educational practices, and that the information was processed such that participants could not be identified (L. Alampay, personal communication, 11 October 2020).

We collected data from 11,736 students, 925 teachers, and 38 non-editing teachers beginning September 9, 2020, and ending on January 9, 2021. The students were undergraduate and graduate students. Undergraduate students were from 18 to 22 years old, while graduate students were 23 and older. Students generally came from middle- to upper-class families. Teachers had at least a bachelor’s degree in the subject area that they were teaching. Most had master’s degrees or higher. Both students and teachers were a mix of males and females, though the exact distribution was not included in the Moodle data.

This time period of data collection represented two distinct academic terms: the first quarter (September 9 to October 24) and second quarter (October 28 to January 9). The dataset contained a total of 2,641,461 logs from 12,699 users. Each transaction was composed of the complete set of the following columns available from Moodle:

- Time—timestamp of the of the action, up to the minute.
- User ID—numerical identifier (ID) of the user performing the action.
- Affected user—numerical identifier of the user affected by the action; When Teacher T sends a notification to Student S, the User ID would be that of Teacher T whereas the Affected user would be Student S.
- Event context—teacher-given name of the module or activity within which the action took place, e.g., “Classroom Exercise 1 Module 1.”
- Component—one of 43 Moodle-defined categories under which various events take place, e.g., Quiz.
- Event name—one of 244 Moodle-defined names for actions that can be performed by the user, e.g., Quiz attempt viewed.
- Description—narrative description of the action performed by the user, e.g., The user with id '1603' has viewed the attempt with id '20202' belonging to the user with id “1603” for the quiz with course module id “18804.”
- Origin—The method used to access Moodle (examples: web, cli (Client), etc.).
- IP address—If Moodle is accessed via the web, this gives the originating IP address (this was anonymized or deleted to ensure data privacy concerns).

The users of Moodle fell into three categories: *teachers*, *non-editing teachers* (e.g., a teaching assistant; non-editing teachers may view and grade work but may not edit or delete course content), and *students*. Because the logs did not include the user category, the university’s systems administrators provided the researchers with each user’s type.

We used transaction log volume, i.e., counts, as the indicator of participation. A transaction is defined as any interaction with Moodle. Each time a student performs an action such as accessing course materials or answering a quiz within Moodle, that action is logged as a transaction. The more the student works within Moodle, the more transaction Moodle logs for that student. While we were interested in broad types of transactions such as quizzes, we did not examine the actual content of course activities and resources. We did not read lectures, discussion postings, exams, quizzes, etc. To answer our research questions, an examination of transaction categories and volumes was sufficient.

## Data preprocessing

The raw data consisted of 3 files of User IDs and User Types (each file representing a user type), and one transaction log file for each of the 123 days of the academic term under study. To preprocess the data, we first merged the list of User IDs and User Types with the transaction logs. We eliminated identifying features such as IP addresses, user full names, and ID numbers. We also had to parse and separate the Time column into separate Date and Time features. The log file was then aggregated according to the Date, User Type, and Component, and the rows that fall under each category were counted. All preprocessed files were then appended to a single file of transactions.

The second phase of the data preprocessing procedure, just prior to the CausalImpact analysis, was to normalize the data (See Table 1). We first aggregated the data frame according to User Type and Component columns (305 for 2020-10-10 and 1133 for 2020-12-23). We took the maximum possible Total for each group across all dates (6146). Then, the items in the Total column were divided by their respective maximum possible value according to the User Type and Component, normalizing the data between 0 and 1 for each User Type and Component (0.05 and 0.18).

**Table 1:** Sample of collapsed data

User Type	Component	Date	Total	Max	Normalized
AS_NON-EDITING-TEACHERS	All Logs	2020-10-10	305	6146	0.05
AS_NON-EDITING-TEACHERS	All Logs	2020-12-23	1133	6146	0.18

We then decided to model three of the top ten most frequently occurring components overall: System, Quiz, and Assignment which, together, represented over 88% of all transactions (See Table 2). System refers to all actions related to communication and course management. Quizzes in Moodle are activities that are completed online and are often automatically graded. Assignment in Moodle is usually file uploads of work completed outside of the LMS.

**Table 2:** Descriptions of the System, Quiz, and Assignment Moodle components

Component	Description	Transaction examples	Percentage of transactions (%)
System	All events related to course communications and management	Course viewed, Course searched, Dashboard viewed, Message sent, User has logged in	63.1
Quiz	All events related to quiz attempts, submissions, creation, and grading	Quiz manually abandoned; Quiz attempt started; Quiz attempt viewed	14.7
Assignment	All events related to the editing, viewing, completion, and grading of assignments	A submission has been submitted; Feedback viewed; Submission viewed	9.6

### CausalImpact analysis

We performed a CausalImpact analysis for four outcome variables: overall student LMS activity, the System component, the Assignment component, and the Quiz component. In this section, we discuss the analysis in three sections: predictor variable selection, time period definition, and CausalImpact results.

### Predictor variable selection

We opted to use teacher and non-editing teacher transactions as predictor variables. Our theoretical grounding for this choice is the teacher expectancy effect (TEE), also known as the Pygmalion Effect. The Pygmalion Effect stems from research on how interpersonal expectations shape reality (Szumski & Karwowski, 2019). It is a form of self-fulfilling prophecy, asserting that teacher expectations have an impact on students’ academic progress. Through verbal and non-verbal behaviors, teachers signal their expectations to students about how the students will (not should) behave or how they will succeed or fail academically (Niari et al., 2016). Students then enact the behaviors or achievement levels that meet teachers’ expectations. Pygmalion effects have been observed at the individual and class level for both achievement outcomes and self-concept (see Friedrich et al., 2015; Szumski & Karwowski, 2019). These effects have been shown to persist over time (see Szumski & Karwowski, 2019). On this basis, we speculate that what teachers signal as their expectations for the online classes will serve as cues to the student about what they will deliver in order to pass the course.

Since teacher and non-editing teacher transactions were categorized under various components, it was necessary to determine which of these components were most predictive. We used Dynamic Time Warping (DTW) to arrive at a parsimonious set of predictor variables. As explained in (Larsen, 2021), the usual approach to finding the relationship between a predictor and a response variable in time series data is to use the Euclidean distance. However, this penalizes instances where the relationships between data have shifted. DTW finds the distance along the warping curve, as opposed to the raw data, to arrive at the best alignment between two time series. We used the MarketMatching R implementation of the DTW algorithm (Larsen, 2021). It should be noted, however, that MarketMatching will only work on predictor variables with a complete set of values and with a variance or standard deviation not equal to 0. To guarantee this, we trimmed the dataset to the top 10 most frequently used components across users. The result of this algorithm was a set of predictor variables with the closest relationship with the response variable (See Table 3).

**Table 3:** Outcome and predictor variables

Student outcome variables	Predictor variables
All Logs	Non-editing teacher System, Teacher File, Teacher Open Forum, Teacher System, Teacher URL
System	Non-editing teacher File, Non-editing teacher System, Non-editing teacher URL, Teacher File, Teacher System
Quiz	Non-editing teacher File, Non-editing teacher Quiz, Teacher Assignment, Teacher File, Teacher Quiz
Assignment	Non-editing teacher Assignment, Non-editing teacher System, Teacher Open Forum, Teacher System, Teacher URL

### Time period definition

**Fig. 1:**
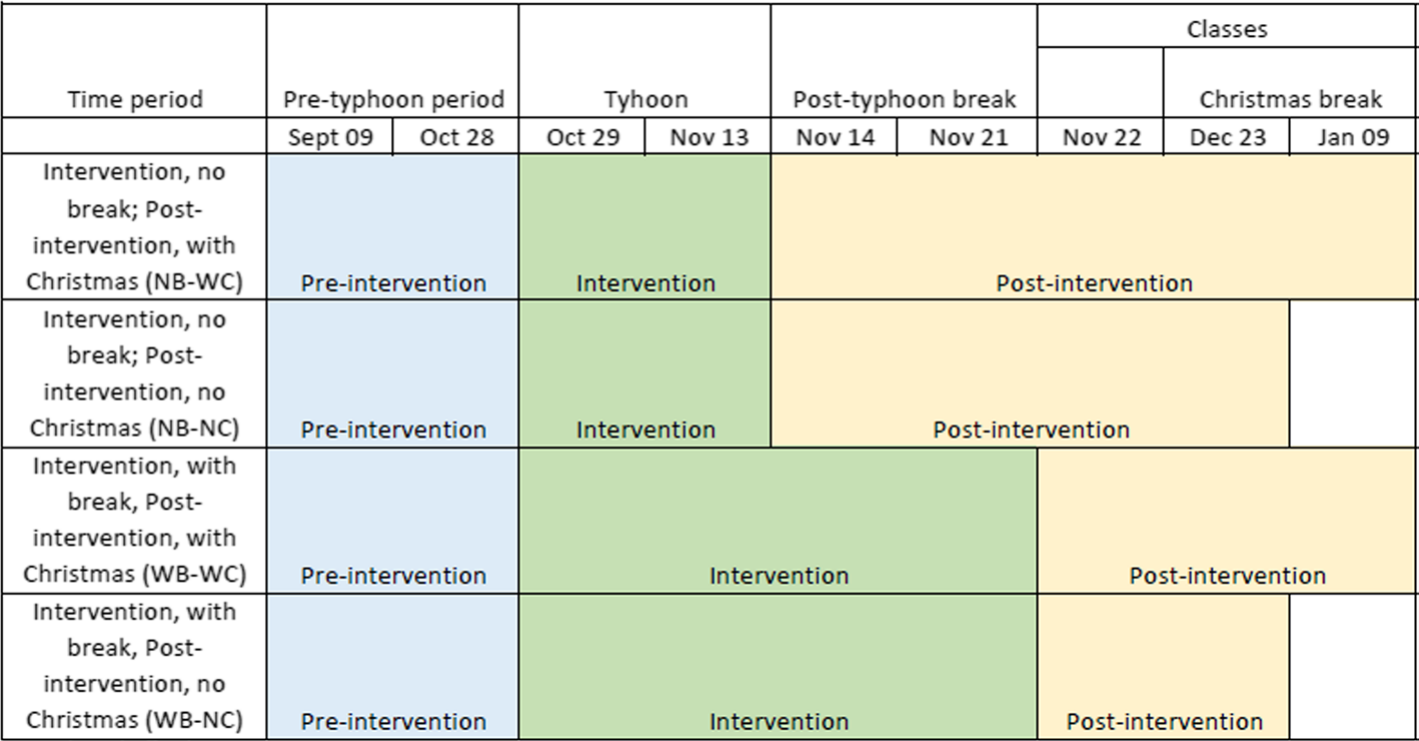
Time period definitions

The definitions of the pre- and post-intervention periods required some consideration. As mentioned in Sect. 2, we collected data from the first quarter (September 9 to October 24) and second quarter (October 28 to January 9) of the academic year. The start of the second quarter was immediately disrupted by Typhoons Goni and Vamco. This led the university to suspend second quarter classes from November 16–21. The university mandated asynchronous-only classes from November 23–28 and resumed synchronous classes, if teachers chose to hold them, from November 29 onward (Vilches, 2020). Furthermore, the second quarter included a Christmas break from December 24 to January 3. To factor in the possible impacts of the class suspension and the Christmas break, we decided to run CausalImpact on four different time periods (See Fig. 1). The pre-intervention period was from September 9 to October 28, the days before the typhoons entered the Philippine Area of Responsibility (PAR). We included October 25 to 27, the period in-between the quarters, because it was during this time that teachers began contacting students to send them links to the online classroom where they would meet on the first meeting day. The intervention period was the period in which the two typhoons struck. We considered two possible endings to this period: November 13, the day Typhoon Vamco left the PAR, and November 21, the last day of the post-typhoon break. The post-intervention period followed and, like the intervention period, had two possible end dates: December 23, before the Christmas break, and January 9. Hence, we created four time periods:

- Intervention period that does not include the post-typhoon break; post-intervention period that includes the Christmas break (NB-WC)
- Intervention period that does not include the post-typhoon break; post-intervention period that does not include the Christmas break (NB-NC)
- Intervention period that includes the post-typhoon break; post-intervention period that includes the Christmas break (WB-WC)
- Intervention period that includes the post-typhoon break; post-intervention period that does not include the Christmas break (WB-NC)

Note that we were working with the same dataset reported in Lagmay and Rodrigo (2021). In this current paper, though, the end date of the intervention period and the start date of the post-intervention periods in time periods WB-WC and WB-NC are different. The dates in Fig. 1 are consistent with the university memo regarding the post-typhoon period (Vilches, 2020). These same time period definitions in Lagmay and Rodrigo (2021) were off by 2 days.

### CausalImpact results

Tables 4 and 5 show the results of the analysis for each of the time periods.

**Table 4:** CausalImpact results

Time period	Posterior inference	All LMS activity		System	
		Actual	Cumulative	Actual	Cumulative
NB-WC	Actual	**0.17**	**9.61**	0.097	5.621
	Prediction (SD)	**0.21 (0.018)**	**11.98 (1.063)**	0.13 (0.018)	7.63 (1.044)
	95% CI	**[0.17, 0.24]**	**[9.97, 14.10]**	[0.096, 0.17]	[5.554, 9.60]
	Absolute effect (SD)	**−** **0.041 (0.018)**	**−** **2.372 (1.063)**	− 0.035 (0.018)	− 2.013 (1.044)
	95% CI	**[−** **0.08, −** **0.006]**	**[−** **4.49, −** **0.36]**	[− 0.069, 0.0012]	[− 3.981, 0.0676]
	Relative effect (SD)	**−** **20% (8.9%)**	**−** **20% (8.9%)**	− 26% (14%)	− 26% (14%)
	95% CI	**[−** **37%, −** **3%]**	**[−** **37%, −** **3%]**	[− 52%, 0.88%]	[− 52%, 0.88%]
NB-NC	Actual	**0.18**	**7.41**	**0.11**	**4.43**
	Prediction (SD)	**0.23 (0.018)**	**9.25 (0.743)**	**0.15 (0.017)**	**6.30 (0.710)**
	95% CI	**[0.19, 0.26]**	**[7.83, 10.77]**	**[0.12, 0.19]**	**[4.96, 7.71]**
	Absolute effect (SD)	**−** **0.045 (0.018)**	**−** **1.843 (0.743)**	**−** **0.046 (0.017)**	**−** **1.872 (0.710)**
	95% CI	**[−** **0.082, −** **0.01]**	**[−** **3.360, −** **0.43]**	**[−** **0.08, −** **0.013]**	**[−** **3.28, −** **0.527]**
	Relative effect (SD)	**−** **20% (8%)**	**−** **20% (8%)**	**−** **30% (11%)**	**−** **30% (11%)**
	95% CI	**[−** **36%, −** **4.6%]**	**[−** **36%, −** **4.6%]**	**[−** **52%, −** **8.4%]**	**[−** **52%, −** **8.4%]**
WB-WC	Actual	0.18	9.07	0.1	5.2
	Prediction (SD)	0.21 (0.018)	10.67 (0.904)	0.14 (0.02)	6.97 (0.98)
	95% CI	[0.18, 0.25]	[8.79, 12.39]	[0.098, 0.18]	[4.911, 8.86]
	Absolute effect (SD)	− 0.03 (0.018)	− 1.59 (0.904)	− 0.035 (0.02)	− 1.740 (0.98)
	95% CI	[− 0.066, 0.006]	[− 3.317, 0.287]	[− 0.073, 0.0065]	[− 3.631, 0.3233]
	Relative effect (SD)	− 15% (8.5%)	− 15% (8.5%)	− 25% (14%)	− 25% (14%)
	95% CI	[− 31%, 2.7%]	[− 31%, 2.7%]	[− 52%, 4.6%]	[− 52%, 4.6%]
WB-NC	Actual	0.21	6.87	**0.12**	**4.04**
	Prediction (SD)	0.24 (0.018)	7.94 (0.592)	**0.17 (0.02)**	**5.57 (0.65)**
	95% CI	[0.21, 0.28]	[6.83, 9.13]	**[0.13, 0.2]**	**[4.28, 6.7]**
	Absolute effect (SD)	− 0.03 (0.018)	− 1.07 (0.592)	**−** **0.046 (0.02)**	**−** **1.525 (0.65)**
	95% CI	[− 0.068, 0.0013]	[− 2.256, 0.0418]	**[−** **0.081, −** **0.0072]**	**[−** **2.659, −** **0.2391]**
	Relative effect (SD)	− 13% (7.5%)	− 13% (7.5%)	**−** **27% (12%)**	**−** **27% (12%)**
	95% CI	[− 28%, 0.53%]	[− 28%, 0.53%]	**[−** **48%, −** **4.3%]**	**[−** **48%, −** **4.3%]**

Bold results are statistically significant

**Table 5:** CausalImpact results

Time period	Posterior inference	Assignment		Quiz	
		Actual	Cumulative	Actual	Cumulative
NB-WC	Actual	0.34	19.47	0.16	9.14
	Prediction (SD)	0.38 (0.039)	21.86 (2.242)	0.17 (0.022)	9.76 (1.253)
	95% CI	[0.3, 0.45]	[17.2, 26.13]	[0.12, 0.21]	[7.25, 12.10]
	Absolute effect (SD)	− 0.041 (0.039)	− 2.383 (2.242)	− 0.011 (0.022)	− 0.621 (1.253)
	95% CI	[− 0.11, 0.039]	[− 6.66, 2.234]	[− 0.051, 0.033]	[− 2.965, 1.891]
	Relative effect (SD)	− 11% (10%)	− 11% (10%)	− 6.4% (13%)	− 6.4% (13%)
	95% CI	[− 30%, 10%]	[− 30%, 10%]	[− 30%, 19%]	[− 30%, 19%]
NB-NC	Actual	0.31	12.78	0.18	7.38
	Prediction (SD)	0.38 (0.038)	15.53 (1.572)	0.17 (0.024)	7.14 (0.968)
	95% CI	[0.3, 0.45]	[12.4, 18.56]	[0.13, 0.22]	[5.17, 8.94]
	Absolute effect (SD)	− 0.067 (0.038)	− 2.749 (1.572)	0.0059 (0.024)	0.2425 (0.968)
	95% CI	[− 0.14, 0.0083]	[− 5.78, 0.3402]	[− 0.038, 0.054]	[− 1.561, 2.212]
	Relative effect (SD)	− 18% (10%)	− 18% (10%)	3.4% (14%)	3.4% (14%)
	95% CI	[− 37%, 2.2%]	[− 37%, 2.2%]	[− 22%, 31%]	[− 22%, 31%]
WB-WC	Actual	0.36	18.19	0.18	8.98
	Prediction (SD)	0.38 (0.04)	18.94 (2.01)	0.18 (0.024)	8.81 (1.178)
	95% CI	[0.3, 0.46]	[15.0, 22.78]	[0.13, 0.22]	[6.41, 10.91]
	Absolute effect (SD)	− 0.015 (0.04)	− 0.754 (2.01)	0.0034 (0.024)	0.1689 (1.178)
	95% CI	[− 0.092, 0.064]	[− 4.592, 3.204]	[− 0.039, 0.051]	[− 1.931, 2.570]
	Relative effect (SD)	− 4% (11%)	− 4% (11%)	1.9% (13%)	1.9% (13%)
	95% CI	[− 24%, 17%]	[− 24%, 17%]	[− 22%, 29%]	[− 22%, 29%]
WB-NC	Actual	0.35	11.50	0.22	7.22
	Prediction (SD)	0.38 (0.04)	12.62 (1.32)	0.19 (0.027)	6.19 (0.904)
	95% CI	[0.3, 0.46]	[10.0, 15.28]	[0.13, 0.24]	[4.43, 7.90]
	Absolute effect (SD)	− 0.03 (0.04)	− 1.12 (1.32)	0.031 (0.027)	1.032 (0.904)
	95% CI	[− 0.11, 0.044]	[− 3.78, 1.461]	[− 0.021, 0.085]	[− 0.677, 2.789]
	Relative effect (SD)	− 8.9% (10%)	− 8.9% (10%)	17% (15%)	17% (15%)
	95% CI	[− 30%, 12%]	[− 30%, 12%]	[− 11%, 45%]	[− 11%, 45%]

No results are statistically significant

#### All LMS Activity

During time period NB-WC, all LMS activity decreased significantly (*p* = 0.02). The response variable had an average value of 0.17. The counterfactual prediction was 0.21. The typhoons therefore had an estimated effect of − 0.041 with a 95% confidence interval of [− 0.077, − 0.0063]. When the data points during the intervention period are summed, the response variable had an overall value of 9.61. The counterfactual prediction was 11.98 with a 95% confidence interval of [9.97, 14.10]. This means that overall student participation decreased by − 20% with a 95% confidence interval of [− 37%, − 3%].

**Fig. 2:**
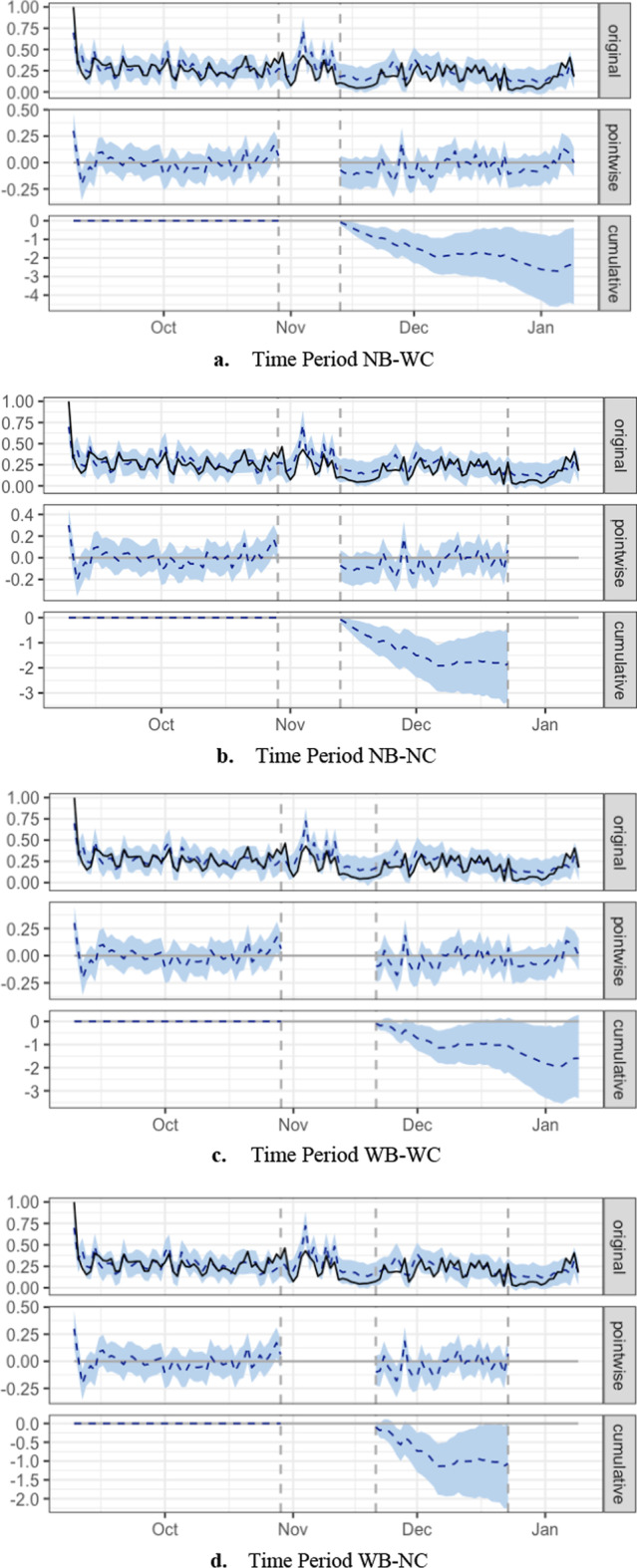
CausalImpact graphs for all LMS activity

Figure 2a shows the CausalImpact graph of all LMS activity for time period NB-WC. Each unit on the *x*-axis represents one day in the time period. The topmost graph labeled “original” shows a solid line representing the actual observed data, i.e., the number of transactions per day. The broken line represents the prediction. The light blue band represents the confidence interval of the prediction. The middle graph labeled “pointwise” shows the difference between the predicted number of transactions and the actual number of transactions per day. If the predicted number of transactions for day 1 was 100 and the actual number of transactions was 80, the pointwise difference was 20. Finally, the cumulative graph at the bottom shows the accumulated difference between the predicted number of transactions and the actual number of transactions. If the pointwise difference on day 2 was 10, the accumulated difference of days 1 and 2 is 30. If the pointwise difference on day 3 was 12, the accumulated difference of days 1, 2, and 3 is 42. The gap in the pointwise and cumulative graphs is the intervention period. There is no accumulated difference during the pre-intervention period. The differences are accumulated post-intervention. Note that the cumulative graph shows a downward trend during the post-intervention period and that there was indeed a slump in the week or so following the typhoons.

During time period NB-NC, all LMS activity also decreased significantly (*p* = 0.01). Student participation had an average value of 0.18. The counterfactual prediction was 0.23. The typhoons therefore had an estimated effect of − 0.045 with a 95% interval of [− 0.082, − 0.010]. When the data points during the intervention period are summed, the response variable had an overall value of 7.41. The counterfactual prediction was 9.25 with a 95% confidence interval of [7.83, 10.77]. Like time period NB-WC, student participation decreased by − 20% with a 95% confidence interval of [− 36%, − 5%]. Figure 2b shows the CausalImpact graph for time period NB-NC.

The results for all LMS activity during time periods WB-WC and WB-NC were insignificant. Time period WB-WC yielded a *p* value of 0.044, while time period WB-NC yielded a *p* value of 0.033. However, in both cases, the signs of the 95% CI fluctuated, which means that even if the *p* value implies significance, the results cannot be meaningfully interpreted. Since time periods WB-WC and WB-NC included the class suspension, it is possible that the definition of the intervention period was too long and the effect of the typhoons had already worn off. Figure 2c, d shows a visualization of this scenario. We trim off the slump that follows immediately after the typhoons. Although the cumulative graph still follows a decreasing trajectory, the difference between the predicted and actual data is no longer significant. Note that the graph shape does not change, regardless of time period. What changes is the size of the intervention period from the end of October to around the middle of November and the length of the graph’s tail.

#### System

**Fig. 3:**
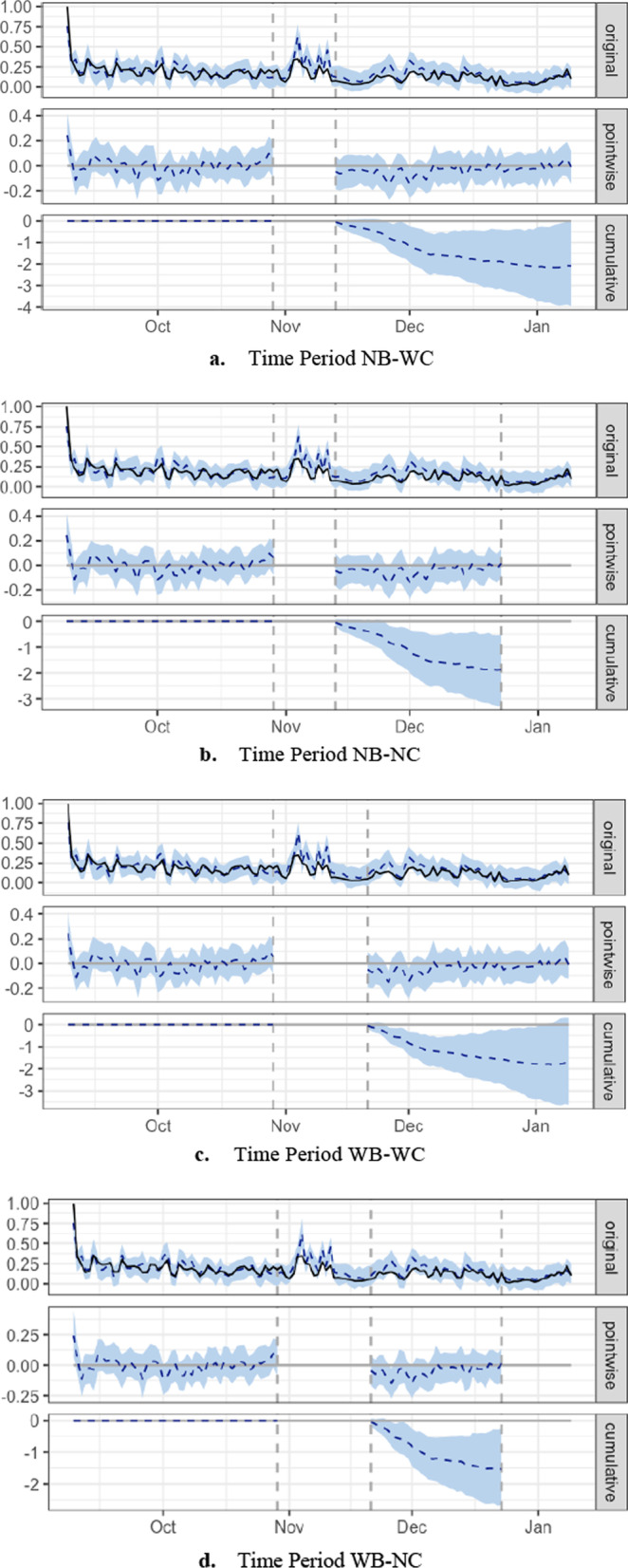
CausalImpact graphs for System component

During time periods NB-WC (Fig. 3a) and WB-WC (Fig. 3c), System activity decreased, but not significantly. Although the *p* value of time period NB-WC was 0.03 and student participation showed a decrease of − 26%, the 95% interval of this percentage was [− 52%, + 1%]. The *p* value of time period WB-WC was 0.04 and the response variable showed a decrease of − 25% with a 95% interval of [− 52%, + 5%]. These fluctuations of the sign during the post-periods of the two time periods meant that the effect is not significant and cannot be meaningfully interpreted (Coqueret & Guida, 2020).

System activity during period NB-NC (Fig. 3b) significantly decreased (*p* = 0.01). Student participation averaged 0.11 as opposed to a counterfactual prediction of 0.15 with a 95% interval of [0.12, 0.19]. The effect of the typhoons is estimated at − 0.046 with a 95% interval of [− 0.080, − 0.013]. The sum of student participation data points during the post-intervention period was 4.43 in contrast to a predicted 6.30 with a 95% interval of [4.96, 7.71].

The results of time period WB-NC (Fig. 3d) were also statistically significant (*p* = 0.01). Student participation averaged 0.12 as opposed to the predicted 0.17 with a 95% interval of [0.13, 0.20]. The effect of the typhoons was therefore estimated at − 0.046 with a 95% interval of [− 0.081, − 0.0072]. The sum of the student participation data points was 4.04 in contrast to a predicted 5.57 with a 95% interval of [4.28, 6.70].

#### Assignments

The effects of the typhoons on student behavior on Assignments were not significant across any of the time periods. *p* values were 0.14, 0.348, and 0.195 for time periods NB-WC, WB-WC, and WB-NC, respectively. Although time period NB-NC had a *p* value of 0.04, student participation’s sign fluctuated. It showed a decrease of − 18% with a 95% interval of [− 37%, + 2%]. This meant that the result could not be meaningfully interpreted.

#### Quizzes

The effects of the typhoons on student behavior on Quizzes were not statistically significant for any of the four time periods. *p* values were 0.29, 0.39, 0.45, and 0.14 for time periods NB-WC, NB-NC, WB-WC, and WB-NC, respectively.

## Discussion

The purpose of this paper was to determine (1) the extent to which extreme weather affected student participation during online classes and (2) whether and at what point they were able to return to pre-typhoon levels of participation. It extends the earlier work by Lagmay and Rodrigo (2021) in several ways: The earlier work only included one time period definition, which we labelled in this paper as NB-NC, while this paper experiments with four different time period definitions. Furthermore, Lagmay and Rodrigo (2021) limited the discussion of the findings to the significance of the decrease, the standard deviation, and the confidence interval. This paper also discusses the absolute and relative effects which were not discussed in the prior paper. Despite these differences in scope, the findings were consistent: Student participation decreased as a whole but those certain components of participation remained at pre-typhoon levels. These findings need to be unpacked for greater nuance.

While student participation as a whole decreased, we found that the significance of the decrease varied, first depending on the definition of the intervention period and second depending on component. When the post-intervention time period excluded the Christmas break (time periods NB-NC and WB-NC), post-typhoon participation as measured in System component significantly decreased, while when the intervention time period excluded the additional week of post-Vamco class suspensions (time periods NB-WC and NB-NC), all LMS Logs significantly decreased.

What was most interesting was that participation in the Assignments and Quizzes components was not significantly different from their predicted behavior, regardless of time period. Because we did not examine the details of actual learning design, course activities, or relative weights of assessments, these findings suggest that students continued to comply with academic assessments as assignments and quizzes make measurable contributions to their grades. System behavior, on the other hand, refers to actions such as checking the course for announcements. These activities are generally not graded. This implies that students were able to continue complying with academic requirements despite the setbacks brought on by the typhoons.

The findings from this study are consistent with findings from prior work on the negative effects of interruptions on academic outcomes. Short-term, small-scale interruptions from social media use, family and friends, sleepiness, and computer malfunctions can derail concentration and throw learning off-course (Zhang et al., 2022; Zureick et al., 2018). Hence, students who experience these interruptions tend to have lower assessment scores than peers who do not. Larger-scale interruptions such as extreme weather and other natural disasters have adverse long-term effects on educational outcomes, especially among marginalized groups (Bernabe et al., 2021; Groppo & Kraehnert, 2017; Marchetta et al., 2018). It is therefore unsurprising that overall student participation dropped following Typhoons Goni and Vamco.

That students were able to continue engaging with Assignments and Quizzes calls for further reflection. How did students still have the capacity to work on assessments when it seemed most logical, under the circumstances, for them to deprioritize their studies in general? The work of Lai et al. (2019) offers some insight in this regard. They found two trajectories of school recovery after a disaster: low-interrupted and high-stable. The low-interrupted trajectory referred to school performance levels that dropped following a disaster, while the high-stable trajectory referred to relatively unchanged performance levels. Schools that had higher levels of attendance in general were more likely to have high-stable trajectories, while schools with a high percentage of economically disadvantaged students were more likely to have low-interrupted trajectories. Sustained engagement with assessments despite the typhoons implies that the university examined in this study had a high-stable trajectory and that its students, by and large, were not economically disadvantaged.

There are solutions available to mitigate the effects of inclement weather. Herrera-Almanza and Cas (2017) studied the long-term academic outcomes of Filipino public school students whose schools were built as part of the Typhoon-Resistant School Building Program of the Philippine government and the Government of Japan. The project made use of Japanese pre-fabrication construction methods and materials to build more structures that were less prone to storm damage, increasing post-typhoon access to schools. The researchers found that students from these beneficiary schools accumulated more years of schooling and were more likely to complete secondary school. Programs such as this illustrate ways in which policy makers can increase the resilience of economically disadvantaged communities.

### Limitations

The generalizability of these findings is subject to at least five limitations. Firstly, CausalImpact analysis requires that the predictor variables should not be affected by the same intervention as the response variable (Brodersen & Hauser, 2014–2017). In this case, it was the likely case that the teachers and non-editing teachers were affected by the typhoons, just as their students were. To this point, we offer two counterarguments: First, we used DTW to find the teacher and non-editing teacher features that were most predictive of student behaviors. The algorithm eliminated the features with no predictive power, leaving only those that could give us a reasonable estimate of student behavior. Multicollinearity was not an issue of concern because CausalImpact’s underlying model “uses spike and slab priors to combat collinearity” (K. Larsen, personal communications, June 29, 2021). The methodology is provided in (Larsen, 2016).

Second, we return to our theoretical framework regarding the Pygmalion Effect (Szumski & Karwowski, 2019). Teacher expectations have been shown to affect student behavior, achievement, and self-concept. Since teachers continued to provide learning materials and assessments after the typhoons and throughout the second quarter, this may have signaled to the students that they were still expected to fulfill their academic obligations.

Our second limitation has to do with the population from which the data were taken. Prior research cited in the “Effects of extreme weather on academic achievement” section showed that extreme weather has detrimental, long-term effects on student achievement, and yet these students seemed to have flourished despite these two typhoons. One possible explanation for this is that the students in this sample were among the best in the country. They generally came from well-to-do socioeconomic backgrounds, and their families had the economic stability to withstand the typhoon’s shocks. Their resilience may not be indicative of the resilience of the Philippines or any developing country as a whole. It may, at best, serve as validation of prior findings that the impact of extreme weather varies along socioeconomic lines. Those who are more financially able will survive, possibly flourish. While it would have been revelatory to perform this analysis on data from an LMS used by less economically fortunate people, such data were not available.

Third, the university had two LMSs working in parallel, Moodle and Canvas. We were only able to capture Moodle data for this study, and the classes using the Moodle server were generally the Computer Science and Management Information Systems classes. The students were therefore technology-savvy and adept at online modes of communication. Students from other courses might have encountered greater challenges.

Fourth, the data captured here represent LMS participation but not other important outcomes such as assessment results, the quality of the educational experience, or the mental health consequences of online learning coupled with severe weather. While students and faculty evidently powered through their requirements, it would be best to triangulate these results with findings and observations from other constituency checks, for a more complete reading of our community.

Finally, as mentioned in the “Time series analysis methods” section, we were not able to consider using CausalTransfer, a more updated version of CausalImpact, nor any of the neural network or deep learning approaches. Future studies may consider experimenting with these other approaches to determine if they yield better results.

Despite these limitations, this paper contributes to technology-enhanced education research and practice. For education researchers, this paper adds to the literature by applying CausalImpact analysis on LMS data to determine the effects of severe weather on students. It contributes to what is quantitatively known about how Philippine students cope with online learning. In the context of severe weather, quantitative research on this subject is still scarce.

This paper serves also as a possible model for researchers who wish to determine the effects of an intervention on a system. They can consider the use of CausalImpact as a possible approach if they have sufficient pre-intervention data for CausalImpact to draw an accurate model, a clearly defined intervention period, and sufficient post-intervention data to serve as a comparison. Future researchers should also be careful with their choice of predictor variables as the behavior of predictor variables should not be affected by the intervention.

For education practitioners, this paper provides evidence that schools and their students can be resilient, and that academic continuity is possible even in the face of difficult circumstances. However, evidence of resilience for some students should not be interpreted as resilience for all. Markers of resilience such as hope and confidence must be grounded in reality (Mahdiani & Ungar, 2021). Resilience should not be used as an excuse for social inequalities and should not shift the responsibility to survive and thrive on people who may lack the power or resources to do so. As extreme weather events that are characteristic to the Philippines, policy makers have to invest in typhoon-resistant infrastructure (Herrera-Almanza & Cas, 2017) and practitioners will have to provide marginalized students with more support in order to achieve desired educational outcomes.

## Data Availability

The dataset(s) supporting the conclusions of this article are available in the RPTEL_CausalImpact_Lagmay_Rodrigo repository, https://github.com/KielLagmay/RPTEL_CausalImpact_Lagmay_Rodrigo. Project name: RPTEL_CausalImpact_Lagmay_Rodrigo. Project home page: https://github.com/KielLagmay/RPTEL_CausalImpact_Lagmay_Rodrigo. Archived version: N/A. Operating system(s): Windows 10 or later (with PowerShell), Windows 8.1 or later (with CMD), macOS 10.13 High Sierra or later (with BASH), macOS 10.14 Mojave or later (with ZSH), or Ubuntu 20.04 or later (with BASH) . Programming language: R, Python, Jupyter Notebook, Shell, PowerShell, and Batch. Other requirements: Anaconda with Python 3.6 or higher + Pyro5, pandas, import_ipynb, netifaces, and dateutil; R with CausalImpact, MarketMatching, dplyr, ggplot2, zoo, tidyr, reshape2, mctest, ppcor, and fsMTS libraries. License: GPL-3.0. Any restrictions to use by non-academics: For data privacy reasons, please send a request to mrodrigo@ateneo.edu for the actual log files, user type files, and aggregated files.
